# A case report of type II Floyd tracheal agenesis with staged tracheal and alimentary reconstructions

**DOI:** 10.1186/s40792-024-02069-4

**Published:** 2024-11-26

**Authors:** Yukiko Tani, Naruhiko Murase, Takazumi Kato

**Affiliations:** 1 Department of Pediatric Surgery, Children’s Medical Center, Japanese Red Cross Aichi Medical Center Nagoya Daiichi Hospital, 3-35 Michishita-Cho, Nakamura-Ku, AichiNagoya, 453-8511 Japan; 2 Department of Gastroenterological Surgery and Pediatric Surgery, Gifu University Graduate School of Medicine, 1-1 Yanagido, Gifu City, Gifu Prefecture 501-1194 Japan

**Keywords:** Pediatric, Tracheal agenesis, Reconstruction, External stenting

## Abstract

**Background:**

Tracheal agenesis is a rare and lethal congenital airway anomaly. In particular, very few patients with type II Floyd tracheal agenesis experience long-term survival. We present the case of a male infant who was diagnosed with type II disease, underwent staged reconstruction and was discharged home with a tolerance for oral intake.

**Case presentation:**

The patient was delivered via emergency cesarean section at 33 week gestation due to fetal distress. Initial attempts to intubate the trachea failed, but ventilation was successfully achieved through esophageal intubation, prompting suspicion of tracheal agenesis with a tracheoesophageal fistula. The diagnosis was confirmed via CT and laryngoesophagoscopy. On the same day, abdominal esophageal banding and gastrostomy were performed. For long-term management, staged operations, including pseudotracheostomy, internal and external stenting of the trachealized esophagus, and reconstruction of the alimentary tract, were performed. Despite being discharged home with a tolerance for oral intake, the patient experienced cardiac arrest at 41 months due to tracheal tube displacement.

**Conclusions:**

Early detection of tracheal agenesis in postnatal patients experiencing respiratory distress is crucial. Type II Floyd tracheal agenesis remains challenging because of the absence of a trachea and the narrow diameter of the tracheoesophageal fistula.

## Background

Tracheal agenesis is an exceedingly rare airway anomaly that occurs in fewer than one in 50,000 live births [1–6]. While polyhydramnios is often observed during fetal development [1, 4, 7], prenatal diagnosis is rare. In cases of respiratory distress soon after birth, accompanied by the absence of crying and difficulty in tracheal intubation, recognizing this rare anomaly and urgently securing ventilation via esophageal intubation are essential for lifesaving measures. Long-term treatment strategies are developed individually on the basis of associated anomalies and the classification of the presence and length of any remaining trachea. However, patients with either type II or III Floyd tracheal agenesis do not have a distal trachea for airway reconstruction, making airway management and long-term survival notably challenging [9]. The aim of this study is to report our experience in treating a patient with type II tracheal agenesis who underwent staged tracheal and alimentary reconstructions and was ultimately discharged home with a tolerance for oral intake, highlighting the importance of early recognition and intervention in managing this complex condition.

## Case presentation

**Fig. 1:**
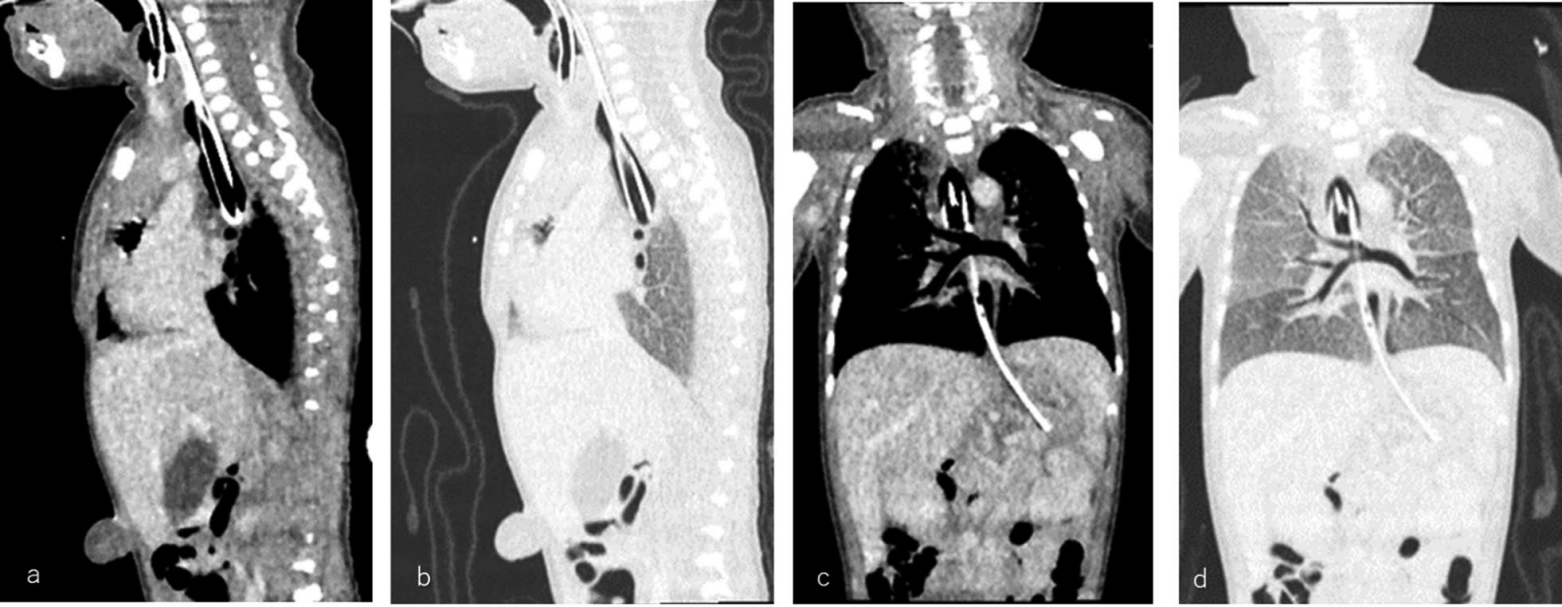
Contrast-enhanced CT imaging. **a, b** Sagittal CT image: two intubation tubes are inserted, one into the larynx and one into the esophagus. **c, d** Coronal CT image: ventilation was successful via esophageal intubation, with the tip of the intubation tube positioned just above the tracheal carina. The gastric tube inserted into the esophagus was inserted into the stomach

A male infant with a history of polyhydramnios was delivered via emergency cesarean section at 33 week gestation due to fetal distress. He weighed 1476 g. He presented with severe respiratory distress in addition to dyspnea and had Apgar scores of 1 at 1 min and 1 at 5 min after birth. The patient was immediately ventilated with a bag and mask, but attempts at tracheal intubation were unsuccessful. However, ventilation through esophageal intubation partially improved his respiratory status. This raised suspicion of tracheal agenesis with a tracheoesophageal fistula. Contrast-enhanced CT imaging (Fig. 1) revealed subglottic obstruction and complete absence of the trachea. The bronchi were present, and the carina communicated with the esophagus through a narrow fistula. Laryngoesophagoscopy showed his vocal cords but no lumen, confirming type II Floyd tracheal agenesis.

**Fig. 2:**
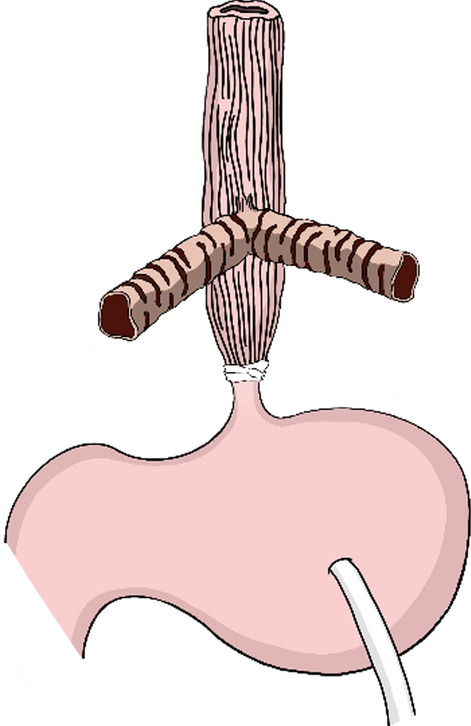
Abdominal esophageal banding and gastrostomy were performed 7 h after birth

Abdominal esophageal banding and gastrostomy were performed 7 h after birth (Fig. 2). Postoperatively, positive pressure ventilation through the tracheoesophageal fistula stabilized his respiratory condition. However, concerns about aspiration pneumonia necessitate further management of the airway, and proper airway management ensures long-term survival.

**Fig. 3:**
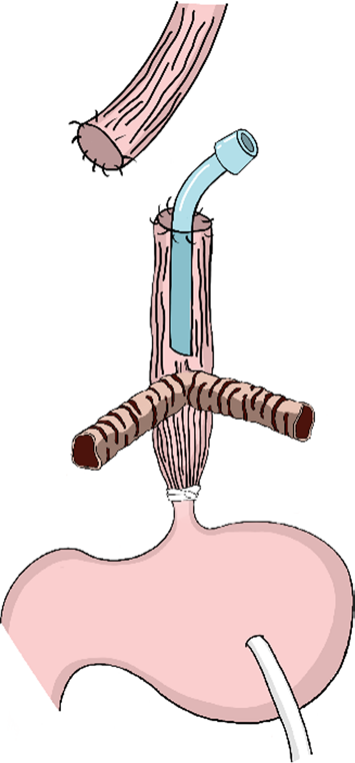
Surgery was performed on day 15 after birth. A cervical esophageal fistula was created. A salivary fistula was created proximally, and a pseudotracheostomy was created distally

**Fig. 4:**
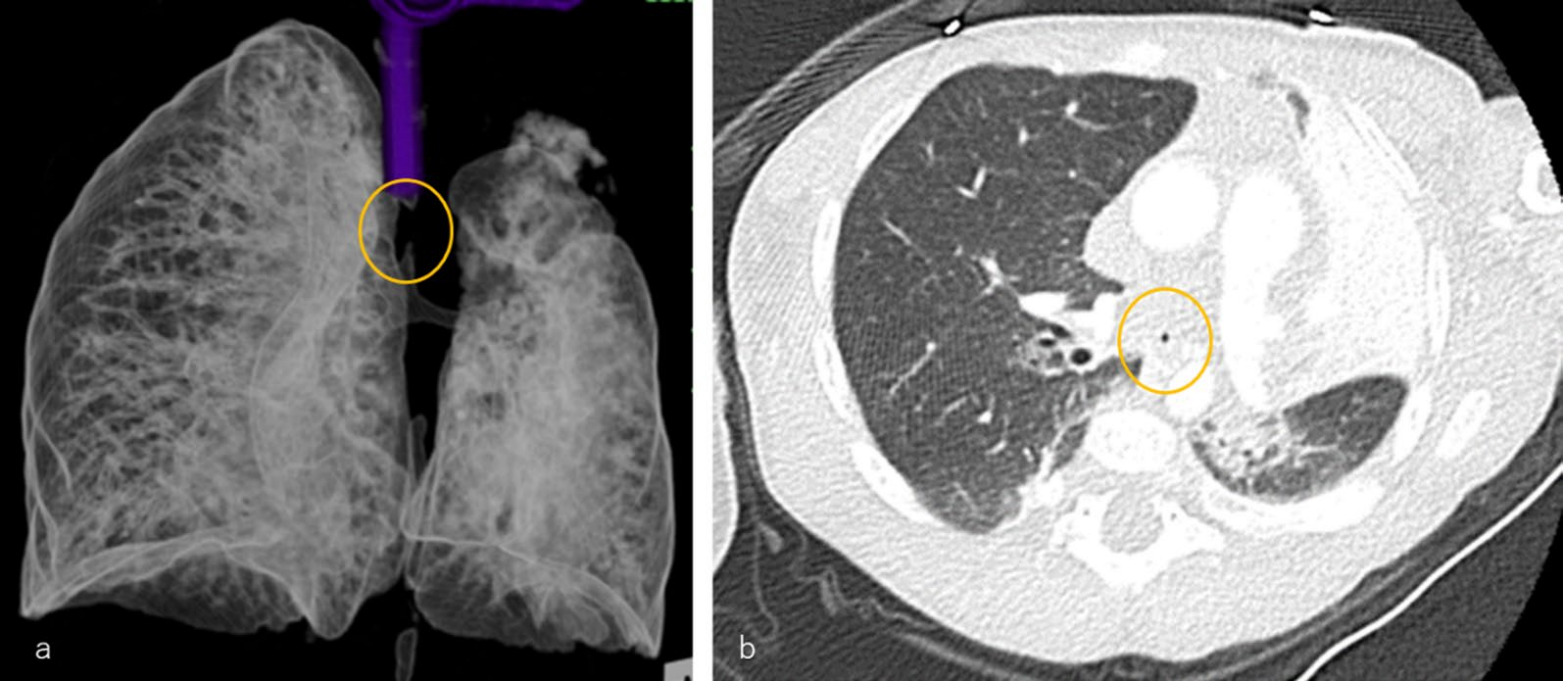
**a** Three-dimensional reconstructed CT image: the tracheoesophageal fistula was pinhole-like and could not be identified. **b** Axial CT image: the tracheoesophageal fistula is pinhole-like and very thin

A second operation (Fig. 3) was performed on day 15 after birth. A horizontal skin incision was made above the sternal notch, and the cervical esophagus was transected at mid-height of the neck. A proximal cervical esophagostomy was performed on the right side for salivary drainage, and a distal cervical esophagostomy was performed for pseudotracheostomy at the midline. An AdjustFit Neo 3.5 mm (Fuji Systems, Tokyo, Japan) was inserted into the distal cervical esophagostomy. Despite these efforts, his respiratory stability became compromised as his weight increased due to the collapse of the trachealized esophagus and the narrow diameter of the tracheoesophageal fistula. Attempts to insert a tube into the tracheoesophageal fistula were hindered by its pinhole-like opening (Fig. 4). Consequently, a customized tracheal tube was fashioned to match the anatomy of the patient's airway at 10 months of age. Its tip was positioned just above the tracheoesophageal fistula to internally stent the esophageal airway and prevent collapse. Upon stabilization of the airway, the trachealized esophagus elongated under positive pressure ventilation, leading to instability of the fixed tube and compromised ventilation over time.

**Fig. 5:**
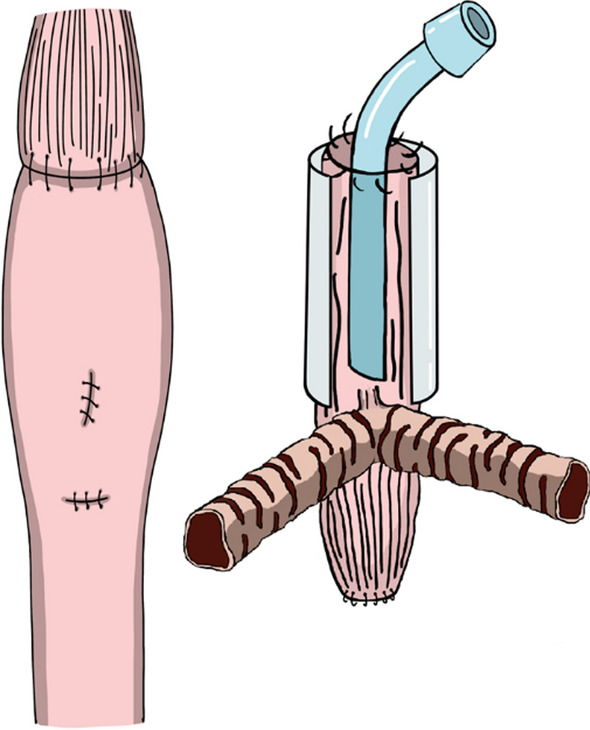
At 14 months of age, external esophageal stenting and reconstruction of the esophagus for alimentary tract function were performed

At 14 months of age, external esophageal stenting and reconstruction of the esophagus for alimentary tract function were performed (Fig. 5). A right thoracotomy was performed through the sixth intercostal space, transecting the thoracic esophagus below the tracheoesophageal fistula. A ringed 16 mm polytetrafluoroethylene graft was fixed around the posterior two-thirds of the trachealized thoracic esophagus for external stenting of the esophageal airway. Owing to the narrow and short tracheoesophageal fistula, external fixation of the stent to the fistula itself was not feasible. Alimentary tract reconstruction involves cervical esophagogastric anastomosis via an intrathoracic route after gastric conduit reconstruction. The distal esophagus, including the site of abdominal esophageal banding, was resected due to severe adhesions. Pyloroplasty was performed using the Heineke–Mikulicz method.

Postoperatively, a stable respiratory status allowed for a reduction in mechanical ventilation settings, and the patient tolerated mechanical ventilation for up to 2 h daily. The patient’s tolerance for oral intake gradually improved following dysphagia rehabilitation. At 16 months of age, the patient was discharged home and the following home mechanical ventilation settings were set: peak inspiratory pressure, 12 cmH_2_O; positive end-expiratory pressure, 5 cmH_2_O; and respiratory rate, 25/min. Unfortunately, the patient experienced cardiac arrest at home at 41 months of age. Abdominal distension at the time of death suggested tracheal tube displacement due to diaphragmatic elevation or dilation of the reconstructed gastric conduit, leading to respiratory distress.

## Discussion

Tracheal agenesis is a serious disorder characterized by more than half of affected infants succumbing within the first week of life and only 7% surviving beyond the first year [1]. This grim prognosis primarily stems from difficulties in promptly addressing postnatal respiratory distress and effectively managing the condition over the long term. Patients with tracheal agenesis typically experience rapid respiratory decline immediately after birth and are unsuitable for tracheal intubation. Consequently, life-saving interventions cannot be effectively implemented without early recognition and understanding of this condition. However, tracheal agenesis occurs is less than one in 50,000 live births [1–6] and is rarely diagnosed in the prenatal period despite the observation of polyhydramnios during fetal development [1, 4, 7]. Owing to the rarity of tracheal agenesis and the difficulties associated with its prenatal detection, early recognition is precluded during initial postnatal respiratory deterioration, potentially contributing to its poor prognosis. Early identification of symptoms such as respiratory distress, the absence of crying, and difficult tracheal intubation is crucial for timely intervention, including esophageal intubation, to improve outcomes in affected patients. Unlike laryngeal atresia, which presents with a similar condition after birth, tracheotomy to secure an airway is not possible, and tracheal agenesis can only be saved by esophageal intubation.

Long-term management necessitates a personalized approach on the basis of the classification of any remaining tracheal structures and associated anomalies. According to Floyd’s classification, tracheal agenesis is categorized into three types: type I, which involves communication between the esophagus and the lower trachea (10–13%), type II, which involves communication between the esophagus and bronchus (59–62%), and type III, which involves separate communications between the esophagus and each main bronchus (23–31%) [8]. While the airway can be immediately stabilized by placing a endotracheal tube in the distal trachea in patients with type I, airway stabilization is difficult in patients with types II and III due to the absence of a distal trachea [9]. Furthermore, the reported diameter of the tracheoesophageal fistula was narrow [6, 12].

Tracheal agenesis frequently cooccurs with other anomalies, notably alimentary tract disorders, which are observed in 80–93.6% of cases [1, 7]. Duodenal atresia and anal atresia commonly occur concomitantly, and associations with congenital heart diseases and renal anomalies suggest a potential link to the VACTERL complex [4–6].

An effective long-term respiratory management strategy begins with abdominal esophageal banding and gastrostomy, followed by cervical esophageal transection and the creation of cervical esophagostomies for pseudotracheostomy and salivary drainage. These treatments can be used for all types of tracheal agenesis. Careful consideration is required when transecting the cervical esophagus to ensure a sufficient proximal esophagus length for alimentary reconstruction while preserving as much of the distal esophagus as possible for future airway reconstruction [10]. Therefore, in our patient, the cervical esophagus was transected at the mid-height of the neck. However, the distal trachealized esophagus expanded and elongated over time under positive pressure ventilation, resulting in compromised tube fixation and ventilation. In addition, the length of the proximal cervical esophagus was insufficient, making extension for esophagoesophageal anastomosis difficult. Therefore, we believe that transecting the cervical esophagus at the lowest possible level would have been beneficial to minimize the amount of dead space in the airway and to facilitate alimentary reconstruction. In this case, the digestive tract was reconstructed using the stomach, but if the cervical esophagus had been resected as low as possible and anastomosis with the abdominal esophagus had been possible, digestive tract reconstruction would have been possible. In addition, because the length of the digestive tract was limited, the posterior mediastinum would have been the optimal route for esophageal reconstruction.

Additional respiratory management methods depend on individual circumstances. While inserting a tube into a tracheoesophageal fistula is an ideal method, its narrow diameter in type II and III cases often poses challenges. In our case, owing to the pinhole-like opening of the fistula, the insertion of a transfistula tube was impossible. Instead, external stenting of the trachealized thoracic esophagus, complemented by internal stenting, was performed. The trachealized esophagus easily collapses, hindering adequate airway maintenance and complicating the clearance of respiratory secretions and the protection of the lung from external stimuli [12]. The use of an external supportive stent with a radical traction suture around the esophagus has been reported to be beneficial for preventing collapse [10, 12].

Although some researchers advocate for tracheoesophageal fistula resection and esophago-tracheal anastomosis [2, 6, 10, 11], this procedure requires cardiopulmonary bypass and poses particular challenges in type II and III cases, where the distal trachea required for anastomosis is absent and postoperative transanastomotic tube fixation is impossible.

Only a few patients who survived tracheal agenesis were able to tolerate oral intake. This limitation is primarily because most of the esophagus is used for airway reconstruction, and alimentary reconstruction is not performed early in life. In our case, oral consumption was possible owing to aggressive alimentary reconstruction and dysphagia rehabilitation. The patient could be discharged home with mechanical ventilation, and the quality of life was relatively good. Unfortunately, however, the patient experienced cardiac arrest at home at 41 months, highlighting the complexity and challenges associated with the long-term management of tracheal agenesis.

## Conclusions

Early recognition of tracheal agenesis in cases of postnatal respiratory distress is crucial for ensuring patient survival. The long-term management of tracheal agenesis requires a multidisciplinary approach involving careful consideration of classification, associated anomalies, and the evolving needs of the growing patient population. Patients with type II and III Floyd tracheal agenesis experience distinct challenges because of the absence of a trachea and the narrow diameter of the tracheoesophageal fistula. Continuous evaluation and adaptation of treatment strategies are essential for optimizing outcomes in these rare and complex cases.

## Data Availability

No data or materials available for case report.
